# A Nanoenzyme Constructed from Manganese and Strandberg-Type Phosphomolybdate with Versatility in Antioxidant and Modulating Conformation of A*β* Protein Misfolding Aggregates In Vitro

**DOI:** 10.3390/ijms24054317

**Published:** 2023-02-21

**Authors:** Jiai Hua, Feng Wang, Xueman Wei, Yuxin Qin, Jiameng Lian, Jianhong Wu, Pengtao Ma, Xiang Ma

**Affiliations:** 1 Chemistry and Chemical Engineering Department, Taiyuan Institute of Technology, Taiyuan 030008, China; 2 Laboratory of Biochemistry and Pharmacy, Taiyuan Institute of Technology, Taiyuan 030008, China; 3 Henan Key Laboratory of Polyoxometalate Chemistry, Institute of Molecular and Crystal Engineering, College of Chemistry and Chemical Engineering, Henan University, Kaifeng 475004, China; 4 State Key Laboratory of Coordination Chemistry, School of Chemistry and Chemical Engineering, Nanjing University, Nanjing 210023, China

**Keywords:** polyphosphomolybdate, protein-misfolding disease, amyloid *β*-peptide (A*β*), conformation modulation, anti-oxidation

## Abstract

Amyloid *β*-peptide (A*β*) misfolding aggregates with *β*-sheet structures and surplus reactive oxygen species (ROS) are both considered to be the culprit of neuronal toxicity in Alzheimer’s disease (AD). Therefore, modulating the misfolding mode of A*β* and inhibiting ROS simultaneous has become an important method for anti-AD. Herein, a nanoscale manganese-substituted polyphosphomolybdate (H_2_en)_3_[Mn(H_2_O)_4_][Mn(H_2_O)_3_]_2_[P_2_Mo_5_O_23_]_2_·14.5H_2_O (abbreviated as MnPM) (en = ethanediamine) was designed and synthesized by single crystal to single crystal transformation method. MnPM can modulate the *β*-sheet rich conformation of A*β* aggregates, and thus reduce the formation of toxic species. Moreover, MnPM also possesses the ability to eliminate the free radicals produced by Cu^2+^-A*β* aggregates. It can inhibit the cytotoxicity of *β*-sheet-rich species and protect synapses of PC12 cells. MnPM combines the conformation modulating ability of A*β* and anti-oxidation ability, which makes a promising multi-funcational molecular with a composite mechanism for the new conceptual designing in treatment of such protein-misfolding diseases.

## 1. Introduction

Protein misfolding diseases are a group of zoonotic diseases [1], in which Alzheimer’s disease (AD) is one of the hot spot models in the research of biochemistry and pharmaceutical chemistry [2]. The aggregation of senile plaques, made up of extracellular amyloid-*β* peptides (A*β*), is the pathological feature of AD [3,4]. A*β* that maintained non-*β*-sheet conformation is a normal membrane peptide metabolized in the human brain [5,6]. Under pathological conditions, for example misfolded templates or abnormal cerebral metal ions, A*β* can misfold to *β*-sheet-rich species, which is the key step to generate toxic A*β* aggregates [7,8,9,10]. In addition, it is well known that the reactive oxygen species (ROS) caused by metal ions-A*β* aggregates is also a causative factor of the neuronal dysfunction [2,10,11]. Hence, simultaneous inhibiting the formation of *β*-sheet-rich A*β* aggregates and the generation of ROS are the effective method to anti-AD [1,2,3,9,10,11].

It is reported that the formation pathway of *β*-sheet-rich species can be modulated [12], in which small molecules such as dopamine, calmidazolium chloride, and platinum complexes can block amyloid fibrillogenesis [13,14,15,16]. Furthermore, polyoxometalates (POMs), a class of metal-oxygen clusters, have also been recognized as modulators against protein misfolding. Dr. Qu et al. reported a series of POMs and nanoparticles as inhibitors of A*β* aggregation [17,18,19]. Dr. Liu et al. interfered with the formation of misfolding-A*β* aggregation by using nanoscale molybdenum containing POM [20]. Our work has focused on the development of complexes with novel structures for anti-protein misfolding. In 2018, we designed a modified POM, {[CoL(H_2_O)]_2_[CoL]_2_[HAs^V^Mo^V^_6_Mo^VI^_6_O_40_]}·2.5H_2_O, which can intervene with the *β*-sheet aggregates through modulating the conformation of A*β* based on non-covalent strategy [21]. Next, two complexes, 2-{2-[(1H-benzoimidazol-2-yl)methoxy]phenyl}benzothiazole and K_10_Na_2_[Ca_6_P_6_O_12_(H_2_O)_6_][PMo_6_O_28_]_2_·24H_2_O, with *β*-sheet-rich conformation modulation activity based on π-π stacking and electrostatic interaction, were synthesized successfully in 2020 and 2021, respectively [22,23]. However, hitherto, all POMs designed above are single-functional modulators which do not have a direct antioxidant effect. Since AD is a multi-pathogenic disease, it is difficult to cure it by only inhibiting a single target [24]. Therefore, molecules that combine functions of modulating A*β* conformation and antioxidants may be more effective in dealing with those thorny problems.

Recently, more and more attention has been paid to POMs, especially nanoscale POMs, since those compounds have advantages such as the ability to cross the blood–brain barrier (BBB) and protein aggregation intervention [25]. Herein we report the structure and multifunctional property of a newly designed manganese-substituted polyphosphomolybdate, (H_2_en)_3_[Mn(H_2_O)_4_][Mn(H_2_O)_3_]_2_[P_2_Mo_5_O_23_]_2_·14.5H_2_O (abbreviated as MnPM, en = ethanediamine). MnPM can not only inhibit the *β*-sheet transformation of A*β* peptide, but can also act as a nanoenzyme to suppress ROS generation, which, as a result, can reduce the toxicity of misfolding A*β* species to cells in vitro. To our knowledge, MnPM represents the first pure inorganic 2-D manganese substituted Strandberg-type polyphosphomolybdate, which possesses both misfolding conformation modulation and antioxidant multi-function.

## 2. Results and Discussion

### 2.1. Characterization of Structure

**Figure 1:**
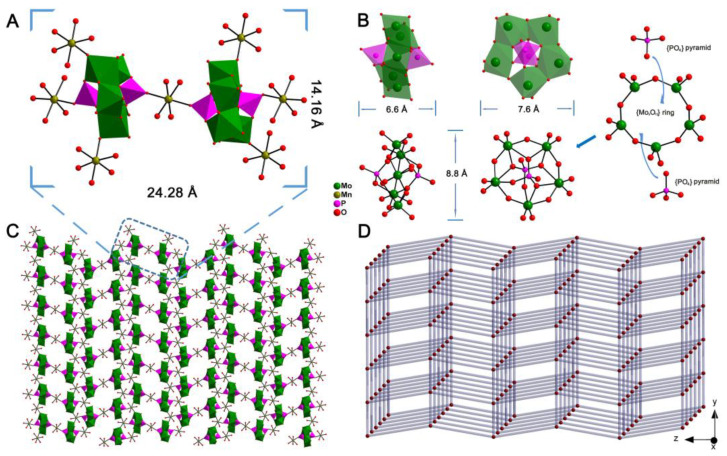
(**A**) Combined polyhedral/ball-and-stick view of the unit of MnPM. (**B**) Polyhedral view and Ball-and-stick representation of one [P_2_Mo_5_O_23_]^6–^ cluster. (**C**) 2D network of MnPM. (**D**) Schematic representation of 4-connected uninodal net of MnPM.

The structure of MnPM was first characterized by single-crystal X-ray diffraction analysis. The selected bond lengths are summarized in Table S1. Detailed information of the single crystal data has been deposited at the Cambridge Crystallographic Data Centre with a CCDC number of 960778. As shown in Figure 1A, X-ray structural analysis reveals that the molecular structural unit of MnPM consists two [P_2_Mo_5_O_23_]^6–^ clusters and seven Mn-complexes [Mn(H_2_O)_4_]^2+^. As shown in Figure 1B, the structure of Strandberg-type [P_2_Mo_5_O_23_]^6–^ cluster can be viewed as a puckered ring of five nearly coplanar corner-sharing/edge-sharing distorted MoO_6_ octahedra [Mo–O: 1.687(3)–2.371(3) Å] with two capping PO_4_ tetrahedra [P–O: 1.501(3)–1.555(3) Å] on both poles of the {Mo_5_O_21_} ring centers. Compared with the free classic Strandberg-type cluster H_6_P_2_Mo_5_O_23_ (more information on the type of POMs, please see Figure S1 and PM.cif in the Supplementary Materials) without coordination with any metal ions, it can be found that the Strandberg-type clusters in MnPM have distortion after coordination with manganese [26]. Those seven manganese complexes can be divided into two categories: 1. one [Mn(H_2_O)_4_]^2+^ located between two Strandberg-type clusters is unique to the unit; 2. The other six [Mn(H_2_O)_4_]^2+^ are divided into two adjacent structural units, each of which accounts for only 0.5 units. Thus, the manganese ions in MnPM have two distinct coordination environments, which is very rare in POM of pure inorganic structure. Finally, as shown in Figure 1C, the units of MnPM interact with each other, resulting in the final 2D framework that is a solid structure. The topological analysis was carried out, and the simplified overall structure of MnPM is a 4-connected uninodal net, as can be seen in Figure 1D with the point symbol of {4^4^·6^2^}.

The bond valence sums (Σs) of oxygen atoms in MnPM were calculated to further study the surface structure characteristics of MnPM [27]. The oxidation states of the oxygen atoms in MnPM were calculated on the following formula:

$$V_{i}=\sum_{j}s_{ij}=\sum_{j}\exp\left(\frac{r_{0}’-r_{ij}}{B}\right)$$

in which *r_ij_* represents the observed values of bond distances listed in Table S1, and *r*_0_′ represents the theoretical value of bond distance between two atoms; The value of *B* was set to 0.37 Å [28]. The theoretical values of Mo–O come from literatures, in which the *r*_0_′(Mo^6+^–O) is 1.900 Å, *r*_0_′(P^5+^–O) is 1.615 Å, and *r*_0_′(Mn^2+^–O) is 1.765 Å [28,29]. As shown in Table 1, the average valence state sum (Σ*s*) of Mo, Mn, and P in MnPM are 6.001, 1.968, and 5.025, respectively.

**Figure 2:**
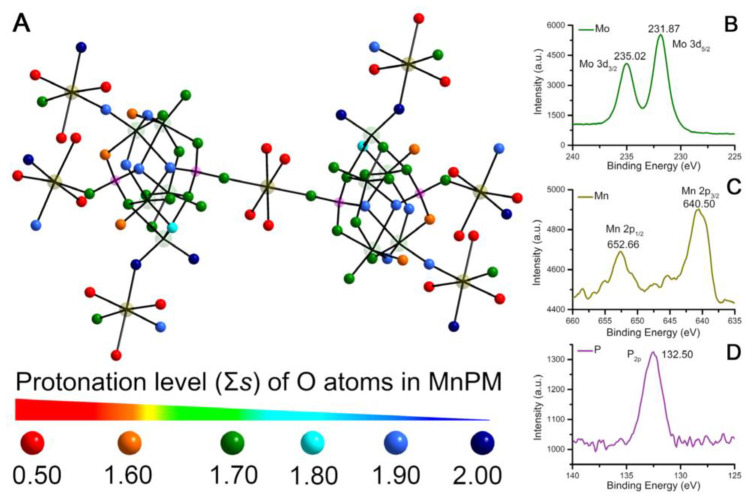
(**A**) Σs of O atoms in the MnPM unit (The extent of Σs for each O atom is indicated by different colors). X-ray photoelectron spectrum (XPS) and the fitted curves of Mo (**B**), Mn (**C**), and P (**D**) in MnPM.

The valence of Mo, Mn, and P in MnPM has been further investigated by using X-ray photoelectron spectrum (XPS). As shown in Figure 2B, there are two broad peaks located at 235.02 and 231.87 eV, which can be assigned to Mo 3d_3/2_ and Mo 3d_5/2_, respectively [30], which may indicate that Mo with +6 exist in MnPM. As can be seen in Figure 2C, the XPS spectrum includes two wide peaks at 640.50 and 652.66 eV that can be assigned to Mn 2p_3/2_ and Mn 2p_1/2_, respectively [31]. These results suggest that the +2 valence Mn ions are present in MnPM. As shown in Figure 2D, there is one peak located at 132.50 eV assigned to P_2P_, which may imply that the valence of P is +5 [32]. Those XPS results are consistent with the results of BVS calculation.

Since the polyanions of POMs have high negative charges and rich basic surface oxygen atoms, they can easily be protonated [33]. The 80 oxygen atoms in MnPM can be classified into four groups: terminal O_t_, bridging O*_μ_*_2_, O*_μ_*_3_, and O*_μ_*_4_. As shown in Figure 2A, the O atoms with Σs of 0~1.60 could act as H-donors owing to the delocalized protons on them, whereas the O atoms with Σs of 1.90~2.00 possess dense electron cloud. As for the remaining O atoms, the electron cloud varies greatly with Σs of 1.60–1.90, which may indicate that they act either as H-donors or H-acceptors. Hence, the O atoms on the surface of MnPM could potentially form H-bonds interacting with peptides. In POM chemistry, the H atoms are usually assigned to be delocalized on the whole fragment, which has been reported in the literature, for example, [Ni(enMe)_2_]_3_[H_6_Ni_20_P_4_W_34_(OH)_4_O_136_(enMe)_8_(H_2_O)_6_]·12H_2_O [27], [H_3_W_12_O_40_]^5–^ [33] and [Cu(en)_2_][Cu(en)_2_H_2_O]_2_{[Cu(en)_2_][Cu_6_(en)_2_(H_2_O)_2_(SiW_9_O_34_)_2_]}·8H_2_O [34].

**Figure 3:**
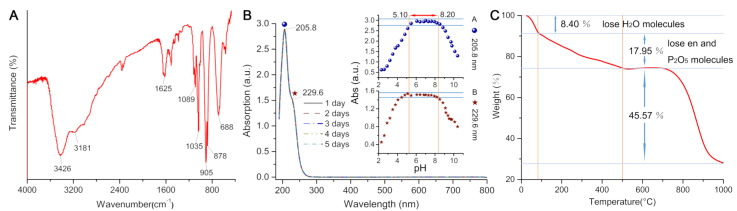
(**A**) IR spectrum for (H_2_en)_3_[Mn(H_2_O)_4_][Mn(H_2_O)_3_]_2_[P_2_Mo_5_O_23_]_2_·14.5H_2_O (MnPM). (**B**) The UV-vis spectrum of MnPM in ultrapure water. Additionally shown: the variation of the peak intensities with pH of the solution (inset figure). (**C**) The thermogravimetric analysis (TGA) curve of MnPM from 25 to 1000 °C.

The IR spectrum of MnPM has also been studied, which has a characteristic asymmetric vibrations to those Strandberg-type cluster containing compounds at low wave-number regions [35]. As shown in Figure 3A, the characteristic bands between 1089–1035 cm^–1^ are attributed to the v(P–O) bond. The peaks between 905 and 878 cm^–1^ are assigned to v(Mo=O) bonds. The prominent bands around 688–550 cm^–1^ are attributed to v(Mo–O–Mo) bond. The occurrence of similar vibrations at about 3181 cm^–1^ may be attributed to the v(N–H) bond, confirming the presence of ligands [36]. A broad feature peak at about 3440 cm^–1^ can be attributed to absorptions of coordinated and lattice water molecules [37].

The stability of MnPM in aqueous solution has further been explored by using UV-vis spectroscopy. As shown in Figure 3B, there are two absorption peaks in the UV-vis spectral data of the aqueous solution, one at 205.8 nm and the other, a shoulder peak, centering on 229.6 nm in the range of 190–500 nm. These two peaks can be assigned to O_t_ → Mo and O*_µ_* → Mo charge transfer transitions, respectively [38]. As can be seen from Figure 3B, MnPM may maintain stability for more than five days in a neutral aqueous solution. As shown in insets of Figure 3B, insignificant variations are noted in the intensity of MnPM UV-vis absorption within a range of pH from 5.10 to 8.20. Out of the range, the absorption peak intensities at 205.8 and 229.6 nm change progressively, which may suggest the commencement of skeletal collapse. The pH range for MnPM stability can therefore be assumed to be from 5.10 to 8.20.

As shown in Figure 3C, the thermogravimetric analysis (TGA) curve of MnPM shows a three-step weight loss. The first step weight loss is 8.40% (cal. 8.29%) in the range 25–85 °C, corresponding to the release of lattice water molecules. The weight loss of the second step is 17.95% (cal. 17.86%) in the range 86–500 °C, corresponding to the release of coordination water and phosphorus pentoxide molecules, which indicates that the framework of MnPM is beginning to collapse [39].

### 2.2. Catalytic Property

ROS are another key species that take responsibility for the neurotoxicity in AD [2,10]. It is reported that Cu^2+^-A*β* can be extremely effective at catalysing ROS, causing damage to neurons [40]. Therefore, a series of antioxidant drugs, such as vitamins, polyphenols, and their derivatives, have been used in research for the treatment of AD [41]. In the last decade, a new type of nanomaterial with enzyme-like characteristics has been developed and known as nanozymes, which has been reviewed in detail by Dr. Wei et al. [42]. In this review, several molybdenum-manganese based nanozymes with special structure have excellent superoxide dismutase (SOD)-like activity [42]. Hence, the effect of MnPM on ROS was investigated by the DCF fluorescence assay.

**Figure 4:**
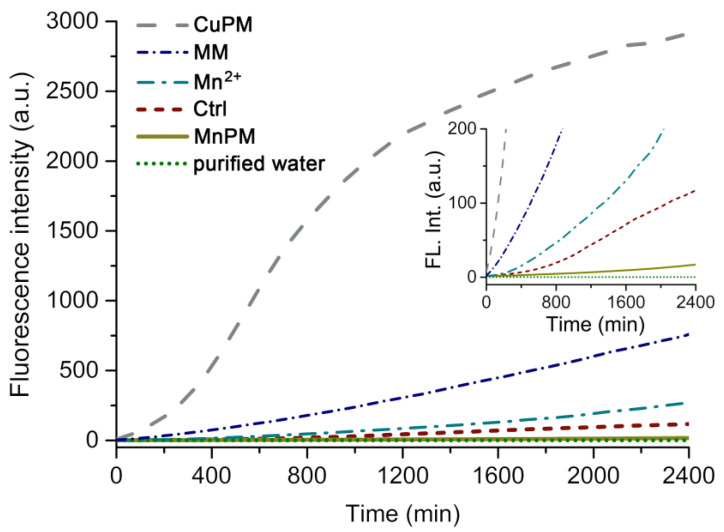
Fluorescence intensity of DCF (*λ*_ex_ = 485 nm, *λ*_em_ = 650 nm) by (H_2_en)_6_[Cu(en)(H_2_O)][Cu(en)(H_2_O)_3_][P_2_Mo_5_O_23_] (CuPM), manganese molybdate (MM), MnCl_2_ (Mn^2+^), blank group (Ctrl), (H_2_en)_3_[Mn(H_2_O)_4_][Mn(H_2_O)_3_]_2_[P_2_Mo_5_O_23_]_2_ (MnPM), and purified water (inset, the expanded graph of the fluorescence intensity in the range of 0 to 200) from 0 to 2400 min.

DCF is a probe derived from non-fluorescent 2′,7′-dichlorofluorecin (DCFH) by the reaction with ROS in the presence of horseradish peroxidase, which can reveal the generation of ROS from the system by special fluorescent emission at 650 nm [43]. As shown in the inset of Figure 4, the DCF fluorescence intensity of MnPM is lower than that incubated with the blank group (Ctrl) from beginning to end, which may imply that the total ROS in the system with MnPM is far less than that without MnPM. Those results may suggest that MnPM possesses SOD-like activities in terms of scavenging free radicals.

Although the experimental group with MnPM alone produced significantly less ROS, the Cu substituted POM (H_2_en)_6_[Cu(en)(H_2_O)][Cu(en)(H_2_O)_3_][P_2_Mo_5_O_23_] (CuPM, see Supplementary Materials CuPM.cif), an isomorphism with MnPM, produces extremely strong ROS, which may indicate that the structure of MnPM did not affect the activity of HRP in the experimental group. Interestingly, as shown in Figure 4, the manganese molybdate (MM, see Supplementary Materials MM.cif) that has the same component as MnPM does not possess those SOD-like capacity, which may indicate that the unique structure of MnPM makes it have antioxidant ability.

### 2.3. Aβ-Peptide Conformational Modulation

**Figure 5:**
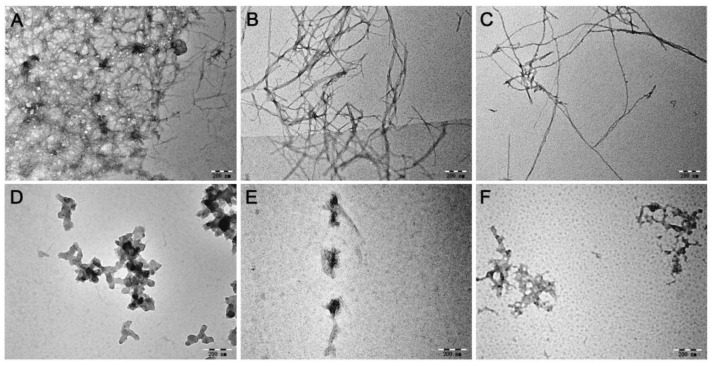
TEM images of A*β*40 (20 μM) in the absence or presence of Zn^2+^, Cu^2+^, and MnPM after incubation at 37 °C and pH 7.4 for 24 h. (**A**) A*β*40 + Zn^2+^; (**B**) A*β*40 + Cu^2+^; (**C**) A*β*40; (**D**) A*β*40 + Zn^2+^ + MnPM; (**E**) A*β*40 + Cu^2+^ + MnPM; (**F**) A*β*40 + MnPM ([A*β*40]:[metal ion]:[MnPM] = 1:2:1) (All the samples with final DMSO content: volume ratio 0.5%).

The morphology of Zn^2+^- or Cu^2+^-incubated with A*β*40 in the presence of MnPM or not were first observed by transmission electron microscopy (TEM). As shown in Figure 5A–C, under Zn^2+^/Cu^2+^ or self-induction conditions, a lot of fibrils can be seen in the groups of A*β*40 + Zn^2+^, A*β*40 + Cu^2+^, and A*β*40, which is the typical characteristic of *β*-sheet-rich protein conformation [44]. The results indicate that abundant soluble *β*-sheet-rich A*β* are in those incubation fluids. However, as can be seen in Figure 5D–F, in the presence of MnPM, the fibrils that symbolize the misfolding peptides all collapsed and formed into amorphous species. The results may imply that the *β*-sheet-rich conformation of A*β* has been destroyed.

**Figure 6:**
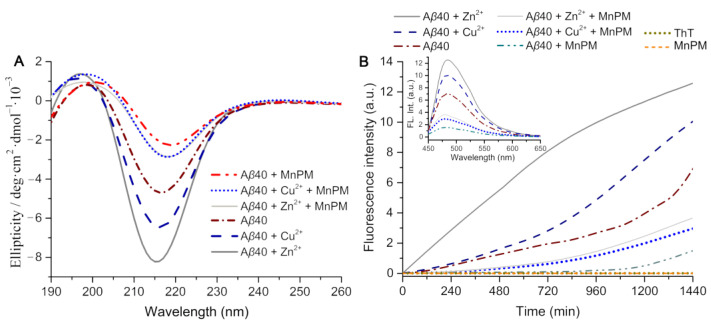
(**A**) CD spectra of A*β*40 (20 μM) and A*β*40 in the presence of Cu^2+^ or Zn^2+^ (40 μM) with or without MnPM (20 μM) after incubation at 37 °C for 24 h (All the samples with final DMSO content: volume ratio 0.5%); (**B**) ThT fluorescence intensity (*λ*_ex_ = 415 nm) of A*β*40 (20 μM) solutions in the absence and presence of Zn^2+^ or Cu^2+^ after incubation with or without MnPM at 37 °C and pH 7.4 for 0–1440 min ([A*β*40]: [metal ion]: [MnPM] = 1:2:1) (inset: ThT fluorescence spectra (*λ*_ex_ = 415 nm) of those A*β*40 fibrils (20 μM) with or without MnPM (20 μM) at 37 °C and pH 7.4 for 24 h).

Circular dichroism (CD) method was further used to verify the effect of MnPM on block the formation of *β*-sheet-related A*β*40 aggregates incubated with Zn^2+^/Cu^2+^. As shown in Figure 6A, a negative band is shown at about 215 nm, which may suggest that the *β*-sheet-rich conformation peptides exist in the Cu^2+^-incubated A*β*40 system [45]. After being incubated with Zn^2+^, the spectrum of A*β*40 + Zn^2+^ group became more negative than that of A*β*40 + Cu^2+^, which suggested that Zn^2+^ can aggravate the *β*-sheet-relate conformational transformation of A*β* peptide [20]. On the contrary, the CD spectra exhibited an obvious recession in negative band after being incubated with MnPM, which may imply that MnPM can suppress the metal-induced *β*-sheet-relate conformational transformation of A*β*. Moreover, the negative band of A*β*40 incubated with MnPM alone is also weaker than that of self-misfolding A*β*40, which may suggest that MnPM can not only suppress the misfolding process by inducing metal ions but also inhibit the self-misfolding of A*β*. Currently, most of the reported mono-functional chelators can only inhibit the conformation misfolding induced by metal ions but do not interact with A*β* per se, which have no effects on self-misfolding A*β* [46]. These results may indicate that MnPM can act as an interfering agent on the formation of *β*-sheets other than chelation mechanism.

The inhibition effect of MnPM on those *β*-sheet-rich fibrils was tested by the ThT assay next. ThT can give rise to a significant enhancement in fluorescence according to the amount of amyloid by specifically binding to the *β*-sheet fibrils [47]. Hence, ThT assay has been widely used to detect the *β*-sheet content in peptide aggregates [21]. As shown in Figure 6B, the fluorescence intensity of A*β* alone group was maintained at a low level before 20 h and began to climb gradually after 20 h, which indicated that A*β* underwent nucleation and rapid transformed into *β*-sheet within 24 h. In the presence of MnPM, the fluorescence intensity of A*β* + MnPM is very weak during the first 16 h. After 16 h, the fluorescence intensity seems to ascend, which implies that increasing amounts of A*β* were transformed into the *β*-sheet. However, the fluorescence intensity of A*β* + MnPM remains weak, indicating that the conformational transformation is largely suppressed. The fluorescence increased obviously when A*β* was incubated with Zn^2+^/Cu^2+^, especially with Zn^2+^, which indicates that Zn^2+^ and Cu^2+^ can promote the formation of *β*-sheet-rich aggregates and that the promotive effect of Zn^2+^ is stronger than that of Cu^2+^. By contrast, in the presence of MnPM, the increasing slope of the fluorescence intensity of Zn^2+^- or Cu^2+^-A*β* solution maintains at a low level, for which the quenching of the fluorescence indicates that MnPM may inhibit the prefibrillar oligomers of A*β* induced by both *β*-sheet misfolding in the presence of metal ions and the self-*β*-sheet-transformation of A*β*. These results are consistent with the conclusion of CD spectrum experiments.

**Figure 7:**
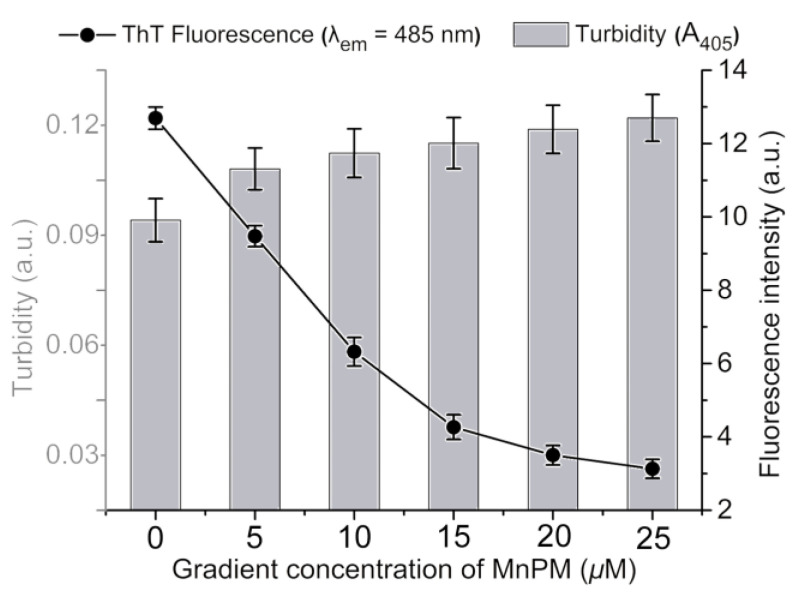
The effect of MnPM on the A*β*40 (20 μM) aggregates after being induced by Zn^2+^ (40 μM) for 24 h determined by ThT assay (*λ*_ex_ = 415 nm, *λ*_em_ = 480 nm) and turbidimetry (All the samples with final DMSO content: volume ratio 0.5%).

Since Zn ions can effectively promote misfolding aggregation process [44], the reverse ability of MnPM on the *β*-sheet of Zn^2+^-A*β* was investigated by the turbidimetry and ThT assay. Turbidity of the solution shows the level of all types of insoluble proteins aggregates [16]. Firstly, A*β*40 was incubated with Zn^2+^ for 24 h to obtain a suspension. Then, different concentrations of MnPM were added to the suspension and incubated for another 24 h to test its effect on those formed *β*-sheet-rich conformation. As shown in Figure 7, on the one hand, the ThT fluorescence intensity had precipitous decline with the addition of MnPM, which may suggest that MnPM can disaggregate the *β*-sheet-rich fibrils of A*β*40. It can be concluded that MnPM not only inhibits the formation of *β*-sheet-rich aggregates, but also has an ability to reverse the formed misfolding *β*-sheet conformation. On the other hand, the turbidity of those solution increases with the addition of MnPM slightly. The result may indicate that MnPM cannot completely reverse the misfolding *β*-sheet-rich A*β* aggregates to its initial state, but aggravates some kind of aggregation probably due to the nucleating effect [48]. It is reported that the content of senile plaques cannot correlate well with the impairment of cognitive function, since a lot of humans who were found to have abundant senile plaques at death did not suffer dementia [49]. Therefore, the aggregates are not only composed of the misfolded *β*-sheet of A*β*, but also contain some other amorphous A*β* aggregates called off-pathway product [14]. The selective induction of A*β* into off-pathway aggregation process is an effective method to suppress the neurotoxicity of *β*-sheet-rich oligomers [45]. Herein, the results demonstrate that some A*β* deposits treated by MnPM are actually not composed of the *β*-sheet aggregates. Hence, the dissociation of the *β*-sheet-related aggregates may suggest that MnPM can eliminate the major neurotoxicity species and may leave the less toxic ones intact in the aggregates.

**Figure 8:**
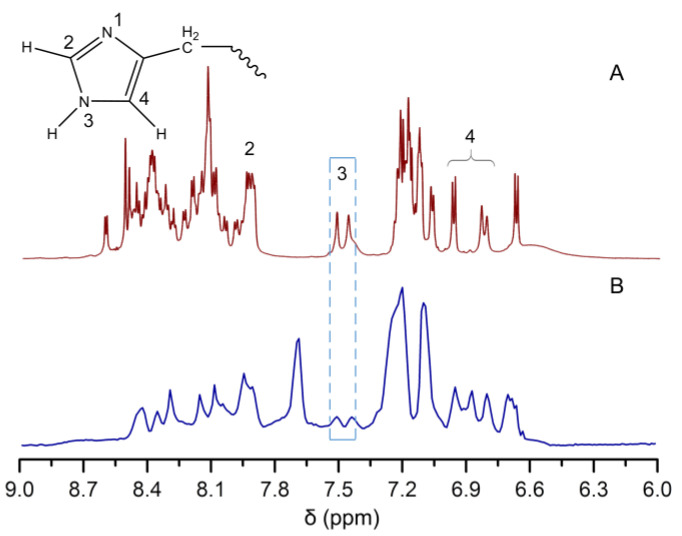
^1^H-NMR spectra of A*β*40 (200 μM) in the absence (**A**) and presence (**B**) of MnPM (200 μM) after incubation at 37 °C and pH 7.4 for 24 h.

Histidine (His−) residues in A*β*40 are the potential metal ion binding sites due to accessible nitrogen donor atoms [16]. Thus, the interactions between MnPM and A*β*40 were further investigated by ^1^H-NMR. As shown in Figure 8A, three signals located at 6.8, 7.5, and 8.0 ppm, respectively, which can be assigned to the imidazole of His-residues, were observed [16]. However, after treated with MnPM, as shown in Figure 8B, the ^1^H-NMR signals of those imidazole changed greatly, in which the H signal located at position 3 is severely attenuated, which may indicate that the His-residues group has coordination with metal ions [47].

### 2.4. Inhibition of Toxicity

**Figure 9:**
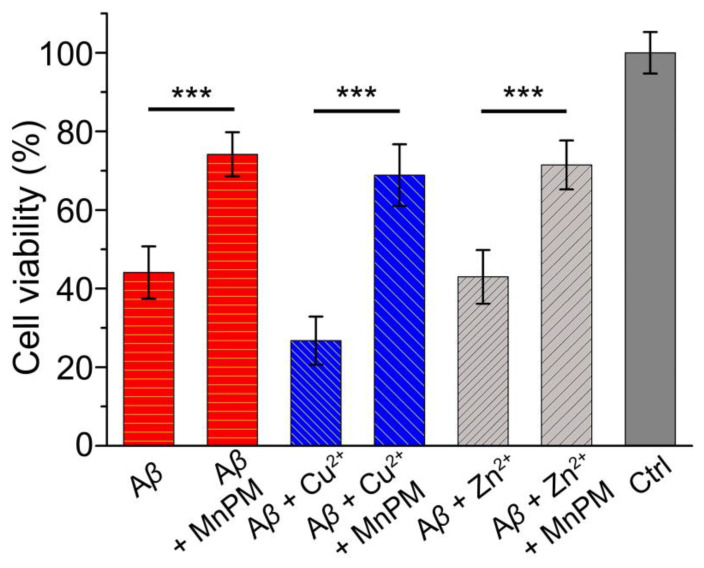
Cell viability of PC12 cells in the presence and absence of A*β*40 with or without Cu^2+^ or Zn^2+^ ions and MnPM determined by MTT assay at 24 h. (A*β*40 = 20 μM, MnPM = 20 μM, [Cu^2+^] = [Zn^2+^] = 40 μM) (All the samples with final DMSO content: volume ratio 0.5%). The results are obtained from three independent experiments and presented as the mean ± standard deviation of the independent experiments. The results were compared using a two-way ANOVA. *** stands for *p* ≤ 0.001.

The toxicity of A*β*40 and metal-induced A*β*40 aggregates with or without MnPM toward neuronal pheochromocytoma (PC12) cells was tested by the MTT assay [50]. As shown in Figure 9, the viability of PC12 cells incubated with Zn^2+^ or Cu^2+^-treated A*β*40 are quite low (<45%), especially A*β*40 + Cu^2+^ group (<30%), which is consistent with the literature report and implies that the metal ions-treated A*β*40 species are highly toxic to PC12 cells [51]. However, in the presence of MnPM, the corresponding cell viability improved significantly, which rose about 35%. Moreover, compared with the group of A*β*40 alone without MnPM, the cell viability of that group with MnPM also increased 25%. In previous literature reports, most of the mono-functional chelators only prevent the formation of *β*-sheet-rich metal-A*β* species from chelating metal ions, but do not possess antioxidant activity [52]. Therefore, MnPM with versatility in antioxidant and modulating conformation of A*β* possesses advantages in inhibiting the toxicity caused by those misfolding aggregates.

**Figure 10:**
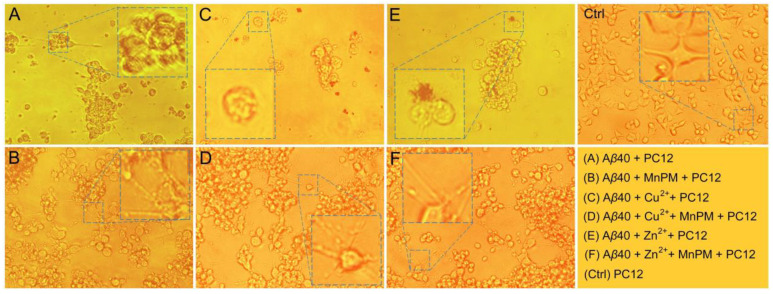
Photomicrographs microscopy images of PC12 cells after incubation with Zn^2+^- or Cu^2+^-induced A*β*40 aggregates with or without MnPM (A*β*40 = 20 μM, MnPM = 20 μM, [Cu^2+^] = [Zn^2+^] = 40 μM) (inset: partial enlarged detail. All the samples with final DMSO content: volume ratio 0.5%).

The effects of MnPM on misfolding A*β*40 aggregates were further investigated by analysis of the morphological changes of PC12 cells under the above conditions. As shown in Figure 10, PC12 cells (control) present polygonal shapes with neurites which have thick, long synapses connected to each other cells, forming a network. However, after incubation with Zn^2+^- or Cu^2+^- or self-misfolding treated A*β*40 aggregates for 1 day, the cells show spherical shapes and the neurites shrank, in which the dendritic networks of neurons were disrupted (as shown in Figure 10A,C,E). Particularly, as shown in the partial enlarged details of Figure 10C, most of the synapses on the cells that were incubated with Cu^2+^-induced A*β*40 aggregates are broken, and the cell body begins to swell. On the contrary, as shown in Figure 10B,D,F, in the presence of MnPM, although there were still deposits in Zn^2+^- or Cu^2+^-A*β*40 group causing the adhesion of the cells, the morphology of cells was maintained as much as possible. These results indicated that enlargement tendency of cell is inhibited. The cell body exhibits spindle shaped mostly, and the synapse is visible clearly. Moreover, the synaptic network of cells is also preserved. It was reported recently that the misfolding A*β* aggregates can cause synaptic toxicity, inducing the injury and dysfunction of neuronal synapses [53]. Therefore, the protection of synapses is also very important and necessary [54]. Those results may imply that MnPM can protect the neurons from synaptic toxicity caused by both metal ions-inducing and self-misfolding A*β* aggregates.

## 3. Materials and Methods

### 3.1. Materials

In this study, reagents used are all of analytical grade, purchased from commercial suppliers, and used as received. Human A*β*40 was purchased from Macklin Agent Ltd. (Shanghai, China), which was verified by HPLC and electrospray ionization mass spectrometry (Supplementary Materials—MS). 3-(4,5-dimethyl-2-thiazolyl)-2,5-diphenyl-2-H-tetrazolium bromide (MTT), Thioflavine T (ThT), 2′,7′-dichlorofluorescin diacetate (DCFH-DA), tris(hydromethyl)aminomethane (Tris) and nerve growth factor 7S (NGF-7S) were purchased from Sigma-Aldrich Inc. (Shanghai, China). Sodium hydroxide (NaOH), hydrochloric acid (HCl), zinc acetate (Zn(OAC)_2_), copper(II) chloride (CuCl_2_), manganese chloride (MnCl_2_), sodium phosphate dibasic dodecahydrate (Na_2_HPO_4_·12H_2_O), ethanediamine (C_2_H_8_N_2_), and sodium molybdenum oxide (Na_2_MoO_4_·2H_2_O) were purchased from J & K Scientific Inc. (Beijing, China). Stock solutions of A*β*40, Zn(OAC)_2_, and CuCl_2_ were prepared according to the reported procedures [16]. The stock suspension of MnPM was prepared by dissolving the compound in DMSO to give a final concentration of 5 mM under ultrasonic concussion before using. All the solutions were prepared with ultrapure water through Milli-Q academic water system and filtered through a 0.22 μm filter (Millipore, Burlington, MA, USA). Pheochromocytoma cells (PC12 cells) were purchased from American Type Culture Collection (ATCC).

### 3.2. Synthesis

Two solutions, A and B, were prepared separately. Solution A: Na_2_MoO_4_·2H_2_O (2.416 g, 10.00 mmol) and Na_2_HPO_4_·12H_2_O (2.399 g, 6.70 mmol) were dissolved in water (30 mL) under stirring. Solution B: MnCl_2_ (1.26 g, 10.00 mmol) and en (0.10 mL, 1.49 mmol) were added to water (30 mL) under stirring. After 10 min, the resulting mixture of B was added to solution A. The mixture was stirred for a further 10 min at room temperature and then the pH value was adjusted to 5.0 by adding 6 mol·L^–1^ HCl dropwise. The solution was kept at 95 °C for 1 h and filtered when it was still hot. The filtrate was allowed to evaporate in an open beaker at room temperature. The colorless transparent crystals had dissolved out of the solution in about 1 week, which can be characterized as Strandberg-type structure compound H_6_P_2_Mo_5_O_23_ [24]. We then sealed the beaker to prevent further volatilization of the solution. After about 3 weeks, the colorless transparent crystals were transformed to yellow crystals (H_2_en)_3_[Mn(H_2_O)_4_][Mn(H_2_O)_3_]_2_[P_2_Mo_5_O_23_]_2_·14.5H_2_O (Yield: ca 29% based on Na_2_MoO_4_∙2H_2_O).

### 3.3. X-ray Data Collection and Structure Refinement

X-Ray data collection and structure refinement: Intensity data of single crystal were collected on a Bruker Apex-2 diffractometer with a CCD detector using graphite monochromatized Mo K*α* radiation (*λ* = 0.71073 Å) at 296 K. Date integration was performed using *SAINT* [55]. Routine Lorentz and polarization corrections were applied. Multiscan absorption corrections were performed using *SADABS* [56]. The structure was solved by direct methods and refined using full-matrix least squares on *F*^2^. The remaining atoms were found from successive full-matrix least-squares refinements on *F*^2^ and Fourier syntheses. All calculations were performed using the SHELXL-97 program package [57]. No hydrogen atoms associated with the water molecules were located from the different Fourier map. The positions of the hydrogen atoms attached to the carbon and nitrogen atoms were geometrically placed. All hydrogen atoms were refined isotropically as a riding mode using the default SHELXTL parameters. A summary of crystal data and structure refinements for MnPM is listed in Table 2.

### 3.4. XPS, IR, UV, and TGA

XPS spectra were conducted on a PHI5000 VersaProbe X-ray photoelectron spectrometer. Elemental analysis was performed on a PQEXCe II ICP-MS. IR/UV spectra were recorded on a NICOLET iS10 and UV-3600 spectrometer, respectively. The TG was tested on a STA449F3 TG-DSC from 25 to 1000 °C.

### 3.5. Catalytic Property

DCF stock solution (1 mM) and horseradish peroxidase (HRP) stock solution (4 μM) were prepared with a Tris buffer (20 mM Tris-HCl/150 mM NaCl, pH 7.4), as described in the reported procedures [58]. The same buffer was used to prepare a 4 µM HRP (horseradish peroxidase) stock solution. All samples were incubated at ambient temperature after adding 10 µM ascorbate that either did or did not contain MnPM (0.025 mM), and then 200 µL of each solution was pipetted into one well of a black 96-well flat-bottomed microplate. DCFH-DA (100 µM) and HRP (0.04 µM) were supplemented, and then the samples were left in the dark at ambient temperature. Fluorescent intensity (*λ*_ex_ = 485 nm, *λ*_em_ = 650 nm) were captured every 10 min from 0 to 2400 min with Thermo Scientific Varioskan Flash microplate reader (Varioskan Flash, Thermo Scientific, Waltham, MA, USA). Spectra of (H_2_en)_6_[Cu(en)(H_2_O)][Cu(en)(H_2_O)_3_][P_2_Mo_5_O_23_] (CuPM), manganese molybdate (MM), MnCl_2_ (Mn^2+^), blank group (Ctrl), (H_2_en)_3_[Mn(H_2_O)_4_][Mn(H_2_O)_3_]_2_[P_2_Mo_5_O_23_]_2_ (MnPM), and purified water were also captured for comparison under the same conditions, as presented above.

### 3.6. Aβ-Peptide Conformational Modulation

#### 3.6.1. ThT Fluorescence Assay

A*β*40 (20 μM) in Tris buffer solution (20 mM Tris-HCl/150 mM NaCl, 990 μL) was incubated with Zn(OAc)_2_ (4 μL, 10 mM) at 37 °C. Following that, MnPM (with the final concentration of 20 μM) or DMSO (final content: 1.5 μL) were added to each sample, respectively, and incubated at 37 °C. Each sample (300 μL) was injected into a well of a flat-bottomed 96-well black plate (Corning Costar Corp). ThT solution (2 μL, 5 mM) was added to each well simultaneously in the dark and incubated at 37 °C. The fluorescence intensity (*λ*_ex_ = 415 nm, *λ*_em_ = 485 nm) was measured by a Varioskan Flash microplate reader (Thermo Scientific) every 10 min from 0 to 1440 min. The fluorescence spectra (*λ*_ex_ = 415 nm) incubated after 24 h was recorded from 450 to 650 nm.

A*β*40 (20 μM) in buffer solution (20 mM Tris-HCl/150 mM NaCl, 992 μL) was incubated with Zn(OAc)_2_ (4 μL, 10 mM) at 37 °C for 24 h. MnPM solutions with the final concentration of 0–25 μM were added to each sample respectively and incubated at 37 °C for another 24 h. All the control groups were treated with DMSO of the same concentration, and the final concentration is 0.5%. The solutions were divided into two parts, one for the ThT assay and the other for the turbidity test. Data were expressed as mean ± standard deviations of at least three independent experiments.

#### 3.6.2. Turbidity Assay

Samples were prepared as described above. Each sample was infused into a well of a flat-bottomed 96-well transparent plate. Turbidity of the solutions were recorded using the absorbance at 405 nm. Data were expressed as mean ± standard deviations of three independent experiments.

#### 3.6.3. Morphological Analysis

Samples were prepared in the same way as ThT fluorescence assay. A drop of solution (10 μL) was spotted on the 300-mesh carbon-coated copper grids at room temperature. After 2 min, the excess solution was removed. The grids were stained with uranyl acetate (10 μL, 1%, *w*/*v*) for 2 min, then they were washed with Milli-Q water (10 μL). The samples were examined on a JEOL JEM-2100 LaB6 (HR) transmission electron microscope.

#### 3.6.4. CD Assay

A*β*40 (20 μM) was dissolved in the Tris buffer solution (20 mM Tris-HCl/150 mM NaCl) and incubated without or with Zn(OAc)_2_ or CuCl_2_ (40 μM) at 37 °C, respectively. MnPM (20 μM) was then dropped to each solution and incubated at 37 °C for 24 h. The CD spectrum of the sample solution was measured on a JASCO J-810 automatic recording spectropolarimeter (Tokyo, Japan) in the range of 190–260 nm. The data acquired in the absence of protein were subtracted from the spectrum. In the control tests, DMSO (final content: 1.5 μL) gave negative results.

### 3.7. ^1^H-NMR

The samples of ^1^H-NMR spectra were prepared by dissolving MnPM (200 μM) in a mixture containing A*β*40 (200 μM), 10% D_2_O, 85% H_2_O, and 5% DMSO-d6, incubated at 37 °C for 24 h, and then centrifuged to get the soluble samples. The ^1^H-NMR spectra were recorded on a Bruker DRX-600 spectrometer.

### 3.8. Inhibition of Toxicity

#### 3.8.1. Inhibition of Toxicity

The PC12 cells used for neurotoxicity, and synaptic dysfunction analysis were prepared as described in the previous literature [59]. The effects of MnPM on the inhibition neurotoxicity were evaluated by using the MTT assay. PC12 cells were incubated with A*β*40 (20 μM) alone or with Zn^2+^- or Cu^2+^(40 μM)-induced A*β*40 complexes in absence or presence of MnPM (20 μM, with final DMSO content: 1.5 μL) for 24 h. Data were expressed as mean ± standard deviations of at least three independent experiments.

#### 3.8.2. Cell Morphological Analysis

The PC12 cells used for this morphological analysis were prepared as above. After incubation for 24 h, the morphological pictures of those cells were captured by a microscope.

Statistical analysis: The results are obtained from three independent experiments and presented as the mean ± standard deviation of the independent experiments. The results were compared using a two-way ANOVA (* *p* ≤ 0.05, ** *p* ≤ 0.01, *** *p* ≤ 0.001).

## 4. Conclusions

It is well-known that the aggregation of misfolded proteins plays a key role in the pathologic pathway of Alzheimer’s disease (AD). Amyloid *β*-peptide (A*β*) in *β*-sheet conformation originated from misfolding aggregation process is the core structure of the toxic species. Furthermore, reactive oxygen species (ROS) derived from those harmful metal-A*β* species is another important neurodegenerative factor. Herein, we described a nanoscale manganese-substituted polyphosphomolybdate (H_2_en)_3_[Mn(H_2_O)_4_][Mn(H_2_O)_3_]_2_[P_2_Mo_5_O_23_]_2_·14.5H_2_O (abbreviated as MnPM) (en = ethanediamine), which possesses both conformational modulation and antioxidant functions. As a conformational modulator, MnPM can prevent the *β*-sheet-rich A*β* aggregation. As a nanoenzyme, MnPM can effectively inhibit and eliminate ROS produced by Cu^2+^-A*β* speices. Thus, MnPM can protect PC12 cells from misfolding A*β* aggregates and ROS-associated toxicity in vitro.

Many other diseases known as prion-like diseases, such as Parkinson’s disease, Huntington’s disease, type-II diabetes, Creutzfeldt–Jacob disease, and new-variant Creutzfeldt–Jakob disease, possess a similar pathogenic processes, which converts the conformation of proteins to *β*-sheet, resulting in large quantities of misfolded proteins and ROS to destroy the brain cells and tissues [60,61]. Since most of them are multifactorial diseases [61], which involve protein misfolding and ROS, the design mechanism of MnPM might be promising and applicative to those protein-misfolding diseases [49,60,61].

## Figures and Tables

**Table 1:** Bond valence and Σs of Mo, Mn, and P in MnPM.

Bond	Valence	Bond	Valence	Bond	Valence	Atom	Σs
Mo(1)–O(1)	2.022	Mo(1)–O(6)	1.700	Mo(1)–O(15)	0.966		
Mo(1)–O(11)	0.841	Mo(1)–O(20)	0.222	Mo(1)–O(16)	1.800	Mo(1)	5.931
Mo(2)–O(2)	1.740	Mo(2)–O(7)	1.722	Mo(2)–O(11)	0.927		
Mo(2)–O(12)	0.905	Mo(2)–O(17)	0.460	Mo(2)–O(20)	0.280	Mo(2)	6.034
Mo(3)–O(3)	1.774	Mo(3)–O(8)	1.653	Mo(3)–O(13)	0.947		
Mo(3)–O(12)	0.885	Mo(3)–O(21)	0.470	Mo(3)–O(17)	0.318	Mo(3)	6.048
Mo(4)–O(4)	1.726	Mo(4)–O(9)	1.631	Mo(4)–O(13)	1.014		
Mo(4)–O(14)	0.987	Mo(4)–O(18)	0.348	Mo(4)–O(22)	0.318	Mo(4)	6.023
Mo(5)–O(5)	1.754	Mo(5)–O(10)	1.698	Mo(5)–O(14)	0.984		
Mo(5)–O(15)	0.902	Mo(5)–O(22)	0.459	Mo(5)–O(16)	0.298	Mo(5)	6.096
Mn(1)–O(19)	0.312	Mn(1)–O(6)	0.273	Mn(1)–O(1W)	0.262		
Mn(1)–O(9)	0.434	Mn(1)–O(2W)	0.164	Mn(1)–O(3W)	0.110	Mn(1)	1.984
Mn(2)–O(23)	0.366	Mn(2)–O(23#)	0.366	Mn(2)–O(4W)	0.320		
Mn(2)–O(4W)	0.320	Mn(2)–O(5W)	0.291	Mn(2)–O(5W)	0.291	Mn(2)	1.953
P(1)–O(19)	1.321	P(1)–O(18)	1.282	P(1)–O(16)	1.225		
P(1)–O(17)	1.202					P(1)	5.030
P(2)–O(23)	1.361	P(2)–O(21)	1.282	P(2)–O(20)	1.202		
P(2)–O(22)	1.176					P(2)	5.021

**Table 2:** Crystallographic data and structural refinements for MnPM.

Empirical formula	C_12_H_146_Mn_6_Mo_20_N_12_O_141_P_8_
Formula weight	5211.61
Crystal system	Monoclinic
Space group	C2/c
*a* (Å)	32.097(9)
*b* (Å)	10.597(3)
*c* (Å)	20.531(6)
*α* (°)	90
*β* (°)	102.695(4)
*γ* (°)	90
*V* (Å^3^)	6813(3)
*Z*	2
*ρ_calcd_* (g·cm^−3^)	2.541
*μ* (mm^−1^)	2.538
*F*(000)	5080
Crystal size*/*mm^3^	0.33 × 0.29 × 0.26
2Θ range for data collection/°	2.03 to 25.00
Index ranges	–38 ≤ h ≤ 22, –12 ≤ k ≤ 12, –24 ≤ l ≤ 24
Rint	0.0202
Data collected	16,875
Independent data	5596
Goodness-of-fit	1.062
Final R indexes [I ≥ 2σ (I)]	R1 = 0.0260, wR2 = 0.0718
Final R indexes [all data]	R1 = 0.0281, wR2 = 0.0731

## Data Availability

Detailed information of MnPM and CuPM has been deposited at the Cambridge Crystallographic Data Centre with a CCDC number of 960778 and 931609.

## Supplementary Materials

The following supporting information can be downloaded at: https://www.mdpi.com/article/10.3390/ijms24054317/s1.
