# Supplementary material for: A Nanoenzyme Constructed from Manganese and Strandberg-Type Phosphomolybdate with Versatility in Antioxidant and Modulating Conformation of Aβ Protein Misfolding Aggregates In Vitro
Source: Int J Mol Sci. 2023 Feb 21;24(5):4317. doi: 10.3390/ijms24054317 (PMC10002135; doi:10.3390/ijms24054317)
Supplement: Supplementary file 1 [file ijms-24-04317-s001.zip › ijms-2143260-supplementary.pdf]

Supplementary Materials

A Nanoenzyme Constructed from Manganese and Strandberg-Type Phosphomolybdate with Versatility in Antioxidant and Modulating Conformation of Aβ Protein Misfolding Aggregates In Vitro

Jiai Hua [1], Feng Wang [1], Xueman Wei [2], Yuxin Qin [1], Jiameng Lian [1], Jianhong Wu [2], Pengtao Ma [3,*], and Xiang Ma [1,4,*]

1 Chemistry and Chemical Engineering Department, Taiyuan Institute of Technology, Taiyuan, 030008, China

2 Laboratory of Biochemistry and Pharmacy, Taiyuan Institute of Technology, Taiyuan, 030008, China

3 Henan Key Laboratory of Polyoxometalate Chemistry, Institute of Molecular and Crystal Engineering, College of Chemistry and Chemical Engineering, Henan University, Kaifeng, Henan 475004, China

4 State Key Laboratory of Coordination Chemistry, School of Chemistry and Chemical Engineering, Nanjing University, Nanjing 210023, China

* Correspondence: mpt@henu.edu.cn (P.M.); maxiang@tit.edu.cn (X.M.) Tel.: +86-351-3569476 (X.M.)

1. Supplementary Figures and Tables

Table S1. Selected bond length (Å) for MnPM.

Mo(1)-O(1)	1.687(3)	Mo(1)-O(11)	1.915(3)	Mo(1)-O(20)	2.261(3)
Mo(1)-O(6)	1.732(3)	Mo(1)-O(16)	2.316(3)	Mo(1)-O(15)	1.879(3)
Mo(2)-O(2)	1.695(3)	Mo(2)-O(7)	1.699(3)	Mo(2)-O(11)	1.928(3)
Mo(2)-O(12)	1.937(3)	Mo(2)-O(17)	2.187(3)	Mo(2)-O(20)	2.371(3)
Mo(3)-O(3)	1.688(3)	Mo(3)-O(8)	1.714(3)	Mo(3)-O(13)	1.920(3)
Mo(3)-O(12)	1.945(3)	Mo(3)-O(21)	2.179(3)	Mo(3)-O(17)	2.324(3)
Mo(4)-O(4)	1.698(3)	Mo(4)-O(9)	1.719(3)	Mo(4)-O(13)	1.895(3)
Mo(4)-O(14)	1.905(3)	Mo(4)-O(18)	2.291(3)	Mo(4)-O(22)	2.324(3)
Mo(5)-O(5)	1.692(3)	Mo(5)-O(10)	1.704(3)	Mo(5)-O(14)	1.906(3)
Mo(5)-O(15)	1.938(3)	Mo(5)-O(22)	2.188(3)	Mo(5)-O(16)	2.348(3)

Mn(1)-O(19)	2.106(3)	Mn(1)-O(6)	2.168(3)	Mn(1)-O(1W)	2.177(3)
Mn(1)-O(9)	2.221(3)	Mn(1)-O(2W)	2.270(3)	Mn(1)-O(3W)	2.286(3)
Mn(2)-O(23)	2.137(3)	Mn(2)-O(23#)	2.137(3)	Mn(2)-O(4W)	2.187(3)
Mn(2)-O(5W)	2.222(3)	Mn(2)-O(5W#)	2.222(3)	Mn(2)-O(4W#)	2.187(3)
P(1)-O(19)	1.512(3)	P(1)-O(18)	1.523(3)	P(1)-O(16)	1.540(3)
P(1)-O(17)	1.547(3)	P(2)-O(23)	1.501(3)	P(2)-O(21)	1.523(3)
P(2)-O(20)	1.547(3)	P(2)-O(22)	1.555(3)		

Symmetry codes: ^{1}1+X,+Y,-1+Z

As shown in Figure S1, the Strandberg-type POM fragment is obviously different from other types of POMs, which there is no derivative relationship from the others. In a brief, the structure of Strandberg-type cluster can be viewed as a puckered ring of five nearly coplanar corner-sharing/edge-sharing distorted MoO_6 octahedra with two capping PO_4 tetrahedra on both poles of the $\{Mo_5O_{21}\}$ ring centers.

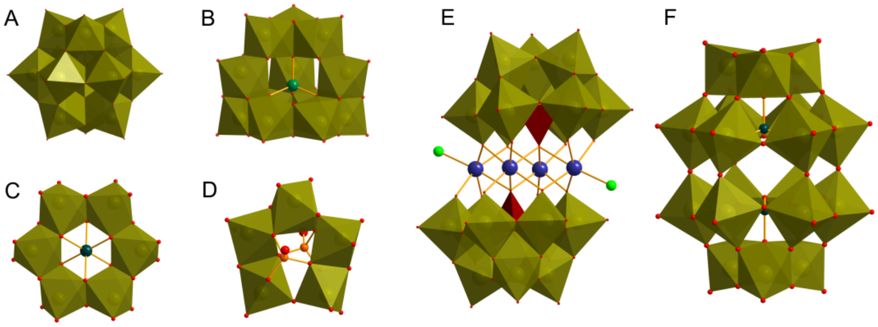

Figure S1. View of classic Polyoxometalate building blocks with bioactive ability. Oxygen atoms: red (The big red balls represent the effective coordination site). (A) α-Keggin-type fragment; (B) ε-Keggin-type fragment; (C) Anderson-type fragment; (D) Strandberg-type fragment; (E) Sandwich-type fragment; (F) Dawson-type fragment.
